# The Wilms’ Tumor Suppressor WT1 in Cardiomyocytes: Implications for Cardiac Homeostasis and Repair

**DOI:** 10.3390/cells13242078

**Published:** 2024-12-17

**Authors:** Sandra Díaz del Moral, Nicole Wagner, Kay-Dietrich Wagner

**Affiliations:** Université Côte d’Azur, CNRS, INSERM, iBV, 06107 Nice, France; sandra.diaz-del-moral@univ-cotedazur.fr (S.D.d.M.); nicole.wagner@univ-cotedazur.fr (N.W.)

**Keywords:** Wilms’ tumor suppressor 1 (WT1), cardiomyocyte, epicardial cells, epicardium, heart, homeostasis, regeneration

## Abstract

The Wilms’ tumor suppressor WT1 is essential for the development of the heart, among other organs such as the kidneys and gonads. The Wt1 gene encodes a zinc finger transcription factor that regulates proliferation, cellular differentiation processes, and apoptosis. WT1 is also involved in cardiac homeostasis and repair. In adulthood, WT1-expression levels are lower compared to those observed through development, and WT1 expression is restricted to a few cell types. However, its systemic deletion in adult mice is lethal, demonstrating that its presence is also key for organ maintenance. In response to injury, the epicardium re-activates the expression of WT1, but little is known about the roles it plays in cardiomyocytes, which are the main cell type affected after myocardial infarction. The fact that cardiomyocytes exhibit a low proliferation rate in the adult heart in mammals highlights the need to explore new approaches for cardiac regeneration. The aim of this review is to emphasize the functions carried out by WT1 in cardiomyocytes in cardiac homeostasis and heart regeneration.

## 1. Introduction

The Wilms’ tumor suppressor WT1 was first identified as a tumor suppressor due to its inactivating mutations in Wilms’ tumor (WT) or nephroblastoma, which is the most common pediatric renal cancer, caused by both excessive cellular proliferation and defective differentiation [1,2,3,4,5]. However, it is now known that only about 5% of nephroblastoma incidence is caused by mutations in WT1; and most Wilms’ tumors exhibit high levels of WT1, which is also the case for most solid cancers as well as leukemia. [6,7,8,9,10,11]. This, in addition to the identification of WT1 as a predictor of acute myeloid leukemia relapse and the fact that its overexpression is related to poor prognosis, has led to the consideration of WT1 as an oncogene [12,13,14,15]. Nowadays, the WT1 protein has been identified as a tumor antigen and is one of the top target molecules for immunotherapy in cancer [14,16,17,18,19].

There are up to 36 different WT1 isoforms based on combinations of alternative transcription and translation start sites, RNA editing and splicing, and post-translational modifications (PTMs) [20,21,22,23]. All isoforms encode a protein that contains four C2H2/Krüppel type zinc fingers that can act as either a transcription factor or an RNA-binding protein [23,24]. Among the plethora of potential isoforms, there are two alternative splicing events, in exon 5 and exon 9, that give rise to the most relevant WT1 isoforms [25]. Alternative splicing of exon 5 generates two isoforms that vary in the presence of 17 amino acids, while the splicing of exon 9 gives rise to isoforms that differ in the insertion of three amino acids, lysine, threonine, and serine (KTS) between zinc fingers 3 and 4 [25]. WT1 isoforms containing the +KTS fragment are involved in RNA processing due to their higher affinity for RNA than DNA. WT1-KTS isoforms bind specifically to DNA, participating in gene transcription, activation, or repression of other genes, depending on the context (reviewed in [22]). The WT1 protein is present in the cell nucleus, but it can shuttle to the cytoplasm, which demonstrates that WT1 functions at different levels of gene expression [26].

The role of WT1 in kidney development, homeostasis, and disease is well-known. However, embryonic *Wt1* deletion is lethal because of cardiac abnormalities, which puts the focus of WT1 function in heart development. Since 1993, it has been demonstrated that mouse embryos carrying the systemic *Wt1* ablation exhibit heart hypoplasia with rounded ventricular apex and thinning of the ventricular walls among other organ abnormalities and die during mid-gestation [27]. Many years later, the epicardial-specific *Wt1* deletion was analyzed using a conditional knockout mouse model, and again, the embryos died presumably due to cardiovascular failure, between embryonic days (E) E16.5 and E18.5 [28]. More recently, conditional *Wt1* deletion in the troponin-T lineage (cardiomyocytes) has been performed, and although it did not cause the death of the embryos, they exhibited severe cardiac malformations that persisted in adult mice [29]. The differences in these mouse models point to the fact that cardiac development and the role of WT1 therein is complex and its function is not restricted to epicardium and epicardial-derived cell types.

In the heart, WT1 expression has been identified in fibroblasts, endothelial, epicardial, and smooth muscle cells, along with the processes it regulates through development and after injury (reviewed in [30]). Although WT1 expression has also been described in cardiomyocytes [31], the functional significance of WT1 expression in this cell type remained unclear until recently. In this review, we summarize the emerging knowledge about cardiomyocyte-WT1 expression in the embryonic and adult heart and the functions that are carried out in homeostasis as well as during regeneration, with the perspective to develop new therapeutic strategies for cardiac repair.

## 2. The Roles of WT1 in Cardiomyocytes in Heart Development and Homeostasis

WT1 is first detected in the mouse at embryonic day 9.5 in the coelomic epithelium and the proepicardium, which is a transitory group of cells that gives rise to the mesothelial lining of the heart, the epicardium [32,33]. A group of WT1-positive proepicardial cells form contacts with the primitive heart tube and proliferate and spread over the myocardial surface to establish the epicardium [32,33,34,35]. Epicardial WT1-expression has been described as being crucial in a subset of cells where it regulates the epithelial-to-mesenchymal transition (EMT), thereby generating epicardial-derived cells (EPDCs) [27,33,34,36,37]. These multipotent mesenchymal progenitor cells later differentiate into fibroblasts, smooth muscle cells, endocardial cells, and cardiomyocytes [34,38,39]. For years, EPDCs were thought to be the only source of the cardiomyocyte lineage [34,40,41], but more recently, a common progenitor pool of the epicardium and the myocardium has been described, providing an additional and independent origin of cardiomyocyte formation [42].

The abnormal epicardium formation, caused by the absence of WT1, in addition to the lethality observed in the epicardial-*Wt1* knockout mouse embryos, led to its consideration as a general epicardial marker [28,33]. Moreover, throughout heart formation, the highest levels of WT1 have been observed in epicardial cells at E12.5 during the EMT, where WT1 regulates the Wnt/β-catenin pathway, and also affects Snai1 and E-cadherin expression [28,36]. In addition, WT1 has been suggested to regulate the retinoic acid system by directly activating RALDH2 in epicardial cells [43], but it was later shown that the retinoic acid system is also activated in cardiomyocytes during development and in response to injury [44]. At later stages of cardiac embryogenesis, WT1 expression decreases, a tendency that is also maintained postnatally [31,33].

**Figure 1:**
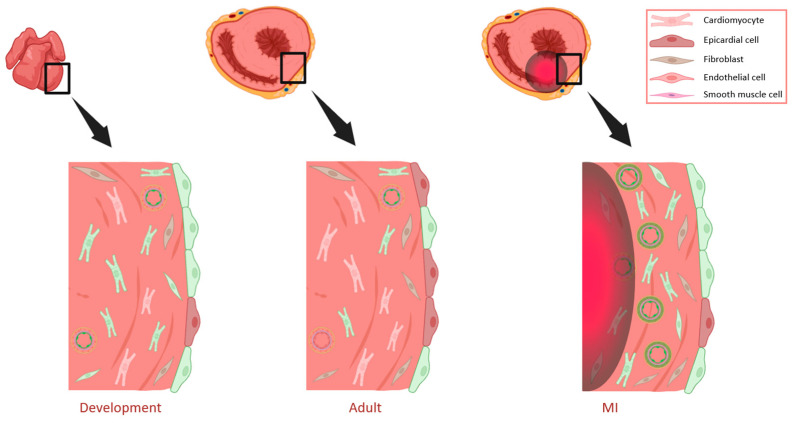
Illustration of WT1 expression in different cardiac cell types in the embryonic, adult, and injured heart. Through cardiac development (**left**), high levels of WT1 are detected in the epicardium, endothelial cells, fibroblasts, and cardiomyocytes. In the adult heart (**central**), its expression is reduced and limited to a subset of epicardial cells, cardiomyocytes, and endothelial cells. After ischemia (**right**), high re-expression of WT1 is observed in the cardiomyocytes, endothelial, epicardial, and smooth muscle cells, mainly located in the border zone of infarction. Cells that express WT1 are colored in green. The infarcted area is represented by the red and black halo. MI: myocardial infarction. Created with BioRender.com.

In the developing mouse heart, WT1 expression is not only restricted to epicardial cells, as it has also been described in endothelial cells, where it is required for the formation of coronary vessels [45,46,47] through activation of the TrkB neurotrophin receptor [45]. WT1 can be detected in developing blood vessels in both the subepicardial area and in the myocardium (Figure 1) [31,45,47,48,49]. Endothelial-specific inducible Wt1-knockout mice have been generated [15], and it has been shown that the developmental deletion of *Wt1* in endothelial cells not only disrupts coronary vessel formation, but also affects myocardial compaction [50]. A very similar observation has been published recently using a different endothelial-specific Cre line [51].

WT1 expression and function in cardiomyocytes remained a controversial issue for a long time, regardless of its detection in some cells years ago [34,52]. A more detailed characterization of cardiomyocyte-specific WT1 expression and function through cardiac development, neonatal stages, and adulthood is summarized in detail in this review. The more recent data are mainly supported by different conditional *Wt1* deletion models in mice and zebrafish models that have been reported for the different developmental time points and adult hearts [29,31,53,54].

Immunohistochemical analyses of embryonic, postnatal, and adult cardiac tissues led to the identification of Wt1 positive cardiomyocytes from developmental day E10.5 until adulthood. As presented in Figure 1, we demonstrate that WT1 is highly expressed in cardiomyocytes during development, and although its expression diminishes postnatally, its expression persists in some cardiomyocytes throughout adulthood [31]. Activation of the Wt1 locus in embryonic cardiomyocytes has also been investigated using Wt1^GFP/+^ knock-in mice [55]. For this purpose, heterozygous Wt1^GFP/+^ knock-in embryos were analyzed at different developmental stages by flow cytometry [29]. Between E10.5 and E11.5, the highest percentage of cardiomyocytes showed activation of the Wt1 locus, as indicated by GFP expression. The percentage was already lower at E14.5 and declined further by E17.5. The immunohistochemistry evaluation of tissue sections from E9.5 and E11.5 embryos confirmed WT1 expression in a fraction of cardiomyocytes. The WT1 signal co-localized to some extended with cardiac troponin T, which was used for cardiomyocyte characterization. Some of these cardiomyocytes were in the wall of the right sinus venosus. In addition, using the lineage tracing model Wt1^Cre/+^;R26R^EYFP^ [39,56], the frequency of cardiomyocytes that had the Wt1 locus activated during cardiac development was also measured. The results confirmed that at stage E12.5, almost one fifth of cardiomyocytes had expressed WT1, with only a slight increase by E15.5, confirming the lower percentage of WT1 expressing cardiomyocytes with progressing development and differentiation [29,31]. Immunofluorescence detection of WT1 in this lineage tracing model revealed that positive cardiomyocytes were mainly located in the left ventricle, the sinus venosus, and the left side of the interventricular septum [29]. Recently, WT1 expression has also been found in embryonic cardiomyocytes from the atria, the compact and the trabecular myocardium of fetal porcine hearts [57], and in cardiac progenitor cells from ischemic human hearts [58].

To explore the functions of WT1 in embryonic cardiomyocytes, a deletion model in the troponin-T lineage was generated (Tnnt2^Cre^;Wt1^Flox/Flox^) [28,59]. WT1 loss of function did not cause embryonic death but increased perinatal mortality [29]. This points to the importance of cardiac WT1 expression in other cell types of the heart such as epicardial and endothelial cells (reviewed in [30]). Embryos with cardiomyocyte-specific *Wt1* deletion showed irregular hearts with severe cardiac malformations from the developmental stage E13.5 onward. The irregularities consisted of abnormal atrium and sinus venosus development, thin ventricular myocardium, less developed or lack of pectinate muscles, and in a lower proportion, defects in the interventricular septum and the cardiac wall. Transcriptomic analysis performed at E13.5 revealed alterations in calcium and potassium handling in embryonic hearts with *Wt1* ablation. This study included the evaluation of adult mice that carried the embryonic *Wt1* deletion. Mutant mice exhibited alterations in electrocardiographic parameters, such as a decreased PR interval and increased QRS and RR intervals, in addition to fibrosis and cardiac anomalies, like the absence of pectinate muscles, aneurism, or even an ectopic muscular septum, among other malformations. The frequency of these cardiac abnormalities was, however, very variable [29].

**Figure 2:**
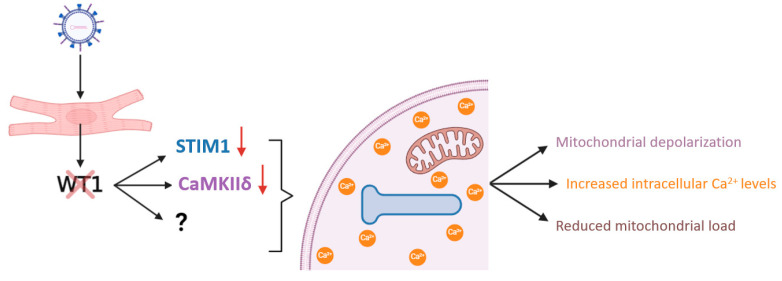
Consequences of in vitro Wt1-silencing in neonatal cardiomyocytes. Cultured neonatal cardiomyocytes were transduced with lentiviral particles expressing NC-RNA (non-coding RNA) as the control, or Wt1 shRNA (short hairpin RNA). Wt1 silencing causes a reduction in STIM1 expression levels, a gene directly regulated by WT1 [60], and CaMKIIδ. This, in combination with the possible additional alteration of other genes that may participate in calcium homeostasis (represented by a question mark), triggers an increase in the intracellular levels of this ion. The lack of WT1 expression in neonatal cardiomyocytes also lowers the mitochondrial content and alters their polarization state. Ca^2+^: calcium. Schematic representation from the Wt1 silencing performed in [53]. Created with BioRender.com.

As already described, although WT1 expression diminishes from embryonic stage E14.5 throughout cardiac development, mRNA expression continues to be detected in the mouse heart after birth, and WT1 continues to be expressed in some cardiomyocytes throughout the lifespan [31]. Cardiomyocytes exhibited WT1 expression in a speckled manner, which leads to the suggestion that the +KTS isoform, which regulates RNA processing, might be involved [31]. In order to identify regulatory functions of WT1 in cardiomyocytes, we performed silencing experiments using ex vivo isolated neonatal cardiomyocytes. Wt1 silenced cardiomyocytes showed alterations in the expression of genes related to calcium and potassium regulation including *Stim1* and *Kcnk2* (also known as Trek-1). Furthermore, the lack of WT1 expression decreased the mitochondrial membrane potential in neonatal cardiomyocytes, and the loss of WT1 also induced variations in the regulation of intracellular calcium levels compared with the control cardiomyocytes (Figure 2) [53].

In cardiomyocytes, calcium regulation is essential for excitation–contraction coupling (ECC), and is also involved in gene expression, among other cellular processes. Alterations in the intracellular levels of calcium can trigger cardiomyocyte malfunction, hypertrophy, and apoptosis [61]. The implication of WT1 in calcium homeostasis is still unclear, although it can be explained by the direct regulation of STIM1 (stromal interaction molecule 1), an activator of the store-operated Ca^2+^ entry (SOCE) [62,63]. Ritchie et al. identified in HEK293 cells that WT1 represses Stim1 expression through binding to a response element in the STIM1 promoter [60]. In contrast, WT1 silencing in neonatal cardiomyocytes led to a reduction in Stim1 expression [53], suggesting a cell-specific regulation. Therefore, the increase in intracellular calcium in neonatal cardiomyocytes silenced for WT1 might be due to the downregulation of STIM1. Low levels of STIM1 would reduce Ca^2+^ stores and cause its accumulation in the cytoplasm, in combination with the also observed diminution of CaMKIIδ (calcium/calmodulin-dependent protein kinase II delta) expression [53]. CaMKIIδ is the major isoform in the heart, and it is known to participate in calcium reuptake through SERCA (sarcoplasmic/endoplasmic reticulum Ca^2+^ ATPase), which is one of the main proteins that resolve cytosolic calcium clearance [64]. However, further research is needed to determine whether WT1 is controlling the expression of other key genes that take part in the regulation of calcium homeostasis in cardiomyocytes, in order to fully elucidate its role.

In the adult heart, WT1 transcripts were initially observed in rat tissues in 1994 [65]. In homeostasis, WT1 expression is reduced compared to the developmental and neonatal stages, and restricted to epicardial cells, some endothelial cells, and a small number of cardiomyocytes in the adult heart (Figure 1) [37].

**Figure 3:**
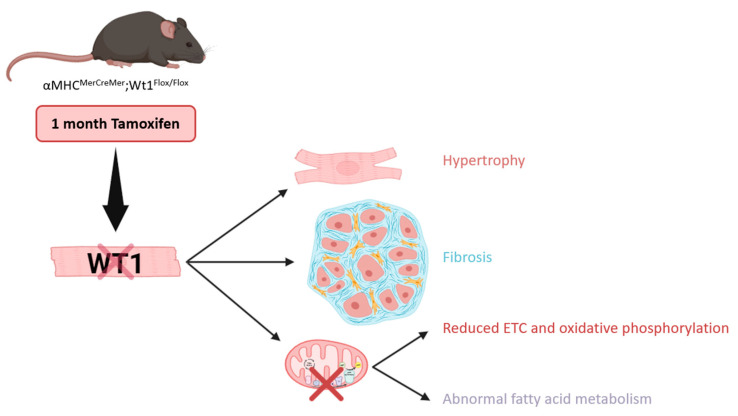
Direct effects of cardiomyocyte specific *Wt1* deletion in adult mice under homeostatic conditions. The transgenic mouse model αMHC^merCremer/+^;Wt1^Flox/Flox^ was used to perform the conditional *Wt1* deletion in adult cardiomyocytes. Compared with the controls, hearts with cardiomyocytic *Wt1* deletion were highly fibrotic. Cardiomyocytes with loss of WT1 function were hypertrophic and showed an abnormal metabolic profile, with a reduction in the proteins involved in fatty acid metabolism as well as in the electron transport chain and oxidative phosphorylation pathways. ETC: electron transport chain. Schematic representation from the *Wt1* ablation assessed in [53]. Created with BioRender.com.

While the roles of WT1 after cardiac injury were postulated many years ago, as will be discussed below, little is known about its functions and the pathways it modulates in the correct functioning of the adult heart. To assess this, conditional *Wt1* deletion in adult cardiomyocytes has been performed, using a αMHC^merCremer/+^;Wt1^Flox/Flox^ transgenic mouse model (Figure 3). This mouse line presents the Cre-recombinase fused to two binding domains of the mutant estrogen receptor (mer), which is inducible by tamoxifen administration, under the control of the α-myosin heavy chain promoter [66]. Fifteen days after tamoxifen administration, a reduction in the QRS interval in mice carrying the *Wt1* deletion could be noted [53]. Proteomic analysis, performed one month after *Wt1* ablation, demonstrated that the lack of Wt1 altered the metabolism of adult cardiomyocytes. This assumption is based on the downregulation of proteins involved in the electron transport chain (ETC) and oxidative phosphorylation pathways, in addition to an abnormal fatty acid metabolism. Flow cytometry analysis from hearts 2 months after tamoxifen ingestion revealed that, as observed in neonatal cardiomyocytes ex vivo, *Wt1* deletion induced mitochondrial dysfunction in this cell type in adult mice. Mutant cardiomyocytes were bigger in size than the controls, however, the heart/body weight ratio was not changed. Picrosirius red staining 2 and 6 months after tamoxifen administration indicated high levels of fibrosis in the mutant animals [53].

Although WT1 is expressed in only a fraction of cardiomyocytes, and its expression levels decrease after birth and remain low in adult mice, its presence is critical for the proper development of the heart and its maintenance in cardiac homeostasis. This notion is based on the consequences observed following its conditional deletion in embryonic, neonatal, and adult cardiomyocytes. The concrete adverse effects resulting from cardiomyocyte specific WT1 loss of function have been masked by the lethality induced through embryonic [27] and adult [67] systemic *Wt1* deletions as well as epicardial ablation [28].

## 3. Re-Expression of WT1 After Cardiac Injury

Myocardial infarction (MI) is the major cause of heart failure (HF), and aside from advances in medical treatment, it is still associated with high mortality [68]. Blockage of a coronary artery, mainly due to the rupture of atherosclerotic plaques, causes a lack of oxygen supply to the myocardium, consequently inducing the death of billions of cardiomyocytes [69]. These lost myocardial cells in the adult heart are replaced by a non-contractile fibrotic scar, and then by ventricular remodeling. This process includes hypertrophy of the surviving cardiomyocytes of the myocardial wall in order to maintain heart function [70] (reviewed in [71,72]).

In light of the pivotal function of WT1 in cardiac development, one of our early studies examined its possible role in cardiac hypertrophy. Analysis of WT1 in left hypertrophied ventricles of spontaneously hypertensive rats (SHRs), animals with transgenic overexpression of the renin-angiotensinogen system as well as in the ventricles of control rats revealed no differences in expression levels. Interestingly, after the induction of MI through ligation of the left anterior descending (LAD) coronary artery, the cardiac WT1 expression levels became rapidly upregulated and remained elevated up to 9 weeks after infarction. Thus, WT1 re-expression does not appear to be related to cardiac hypertrophy but to cardiac ischemia. Through mRNA in situ hybridization and immunohistochemistry, WT1 expression in the epicardium of controls (sham-operated rats) was confirmed. Notably, in addition to its epicardial expression, WT1 signals were significantly elevated in the coronary vessels in proximity to the infarcted region of animals with ligation of the coronary artery. This vascular WT1 de novo expression correlated with the observed increase in the WT1mRNA levels and remained stable up to 9 weeks. Double-immunofluorescent labeling revealed WT1 expression in proliferating endothelial and vascular smooth cells from the inner portion of the myocardial vessels, as represented in Figure 1. Highly interestingly, WT1 expression in coronary vessels after MI could be mimicked by the exposure of animals to hypoxic conditions. This indicates that WT1 is involved in the formation of novel coronary vessels upon myocardial infarction through stimulation by hypoxia, the reduced tissue oxygen supply [73]. WT1 upregulation after MI was confirmed by others in both epicardial and endothelial cells more than a decade later [47].

In the context of myocardial damage, the epicardial cells in the border of the ischemic area lose their integrity 24 h after MI, however, integrity is reestablished three days later [74]. The epicardium, which is quiescent in the adult heart, is reactivated after myocardial injury. It has the capacity to regenerate through the transient re-expression of the embryonic epicardial genes Tbx18, Raldh1, Raldh2, and Wt1, along with proliferation and EPDC formation. As a result, this causes thickening of the space between the myocardium and epicardium [37]. Epicardial WT1 reactivation took place between 1 and 5 days after infarction and its expression diminished 4 weeks later, a time frame similar to Tbx18, Raldh1, and Raldh2 re-expression. Three days after MI, WT1 mRNA was found in the epicardium near the ischemic area; 1 week later, it was detected in the covering of the left ventricle and the apex [74]. Moreover, this WT1 upregulation could be found in up to 75% of the epicardial cells and the EPDCs in the surroundings of the myocardium, while this proportion in the non-injured adult heart was close to 25%, as illustrated in Figure 1 [37].

Our group demonstrated an increase in Wt1-positive cardiomyocytes in hearts from mice with LAD ligation 48 h after MI (acute phase). These cardiomyocytes were mainly located in the border zone of the infarcted area, and they remained WT1-positive along the reparation phase, 3 weeks later (Figure 1) [31]. This points to a potential role of WT1 specifically in cardiomyocytes in cardiac regeneration, in addition to its activation in endothelial and epicardial cells, as described earlier [73].

While the identification of WT1 reactivation in the different cardiac cell types is becoming clearer, the origin of its expression is still controversial. On the one hand, there are several studies supporting that WT1-positive cardiomyocytes differentiate from epicardial progenitors during development [34]. After injury, the WT1-positive activated epicardial cells are a heterogeneous population expressing cardiac progenitor and mesenchymal stem markers [75]. Thymosin β4 priming before infarction resulted in the generation of some epicardial-derived cardiomyocytes after infarction [52,76], while others showed that thymosin β4 priming did not result in the re-programming of epicardial-derived cells into cardiomyocytes [77]. In a different reporter system, a significant increase in Wt1 expression and proliferation in the epicardium shortly after myocardial infarction was observed and the formation of a Wt1-lineage-positive subepicardial mesenchyme cell population described. These cells contributed to fibroblasts, myofibroblasts, and coronary endothelium in the infarct zone, some of them also later differentiated into cardiomyocytes [74]. On the other hand, some investigations showed that, following MI, these cardiomyocytes were not epicardial-derived, but de novo cardiomyocytes originating from stem cells [78]. Interestingly, Tyser et al. characterized, by single cell transcriptomic analyses, a common progenitor cell pool of the myocardium and epicardium. These cells expressed WT1, demonstrating a source for either cardiomyocytes or epicardial cells [42]. Thus, WT1 might be re-activated in both cell types in the adult heart in response to injury.

**Figure 4:**
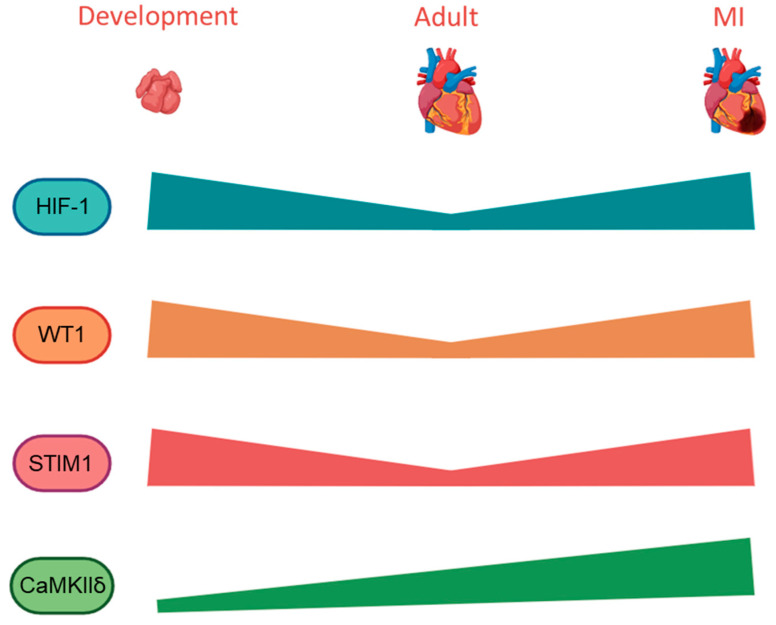
Differential expression pattern of HIF-1, WT1, STIM1, and CaMKIIδ in the heart through development, homeostasis, and injury. The hypoxic environment established in the embryonic heart (**left**), drives high levels of HIF-1, which triggers WT1 expression. At early developmental stages, calcium regulation in the heart is mainly orchestrated by the SOCE pathway, which explains the high levels of STIM1 and low CaMKIIδ expression. In homeostasis (**central**), CaMKIIδ expression starts to increase as its role is more essential, and the STIM1 levels drop in cardiomyocytes after birth. The augmented oxygen availability causes the downregulation of HIF-1, and WT1 expression becomes restricted to a low number of cardiac cells. After ischemia (**right**), not only does HIF-1 expression increase, but the CaMKIIδ levels are also higher compared to homeostasis. Genes highly expressed during embryonic development, such as STIM1 and WT1, are reactivated in the injured heart. MI: myocardial infarction. Created with BioRender.com.

As suggested in the previous section, it should be considered that the cardiomyocyte-WT1 upregulation in the ischemic heart would also trigger the induction of downstream genes related to calcium regulation. For instance, STIM1, which has been identified to be re-expressed in cardiomyocytes during heart failure [79], or CaMKIIδ, whose activity is increased in the failing heart and is associated with the promotion of inflammatory pathways (Figure 4) [80]. The augmented activity of genes that participate in calcium homeostasis could help to reduce the elevated intracellular levels of this ion described in cardiomyocytes after MI [81] (reviewed in [82]), but a deeper evaluation is needed to identify the detailed mechanism.

We were the first to demonstrate that WT1 re-expression after ischemic heart damage is due to hypoxia [83]. We further showed that hypoxic WT1 induction is mediated through the direct transcriptional activation of Wt1 by the hypoxia-inducible factor-1 (HIF-1) [83]. Additionally, the role of HIF-1 [84], as a key element of the epicardial reactivation after injury, is also supported by the regulation of the epicardial invasion it carries out through development [85]. WT1 seems to be additionally activated by hypoxia-inducible factor-2 in some cell lines [86]. Whether this regulation is relevant in the heart remains to be determined. Another possible explanation for WT1 re-expression in the damaged heart could involve soluble factors that are secreted by the myocardium and need to be identified. In the heart, low levels of vitamin D have been associated with the severity of myocardial infarction as well as higher mortality from cardiovascular diseases [87] (reviewed in [88]). The administration of vitamin D in mice and healthy volunteers was shown to stimulate vascular regeneration through HIF-1α induction [89], which in turn would activate Wt1 [83]. This supports the idea that vitamin D could be a promising treatment to improve cardiac health, but clinical trials suggest that vitamin D supplementation does not reduce the risk of cardiovascular events nor the associated mortality [90]. Notably, we demonstrated that the vitamin D receptor (VDR) is a downstream target of WT1. In the presence of the active vitamin D metabolite, WT1 promotes apoptosis in embryonic kidney cells and decreases their proliferation rate through the regulation of VDR [91].

A less frequent cause of HF is due to the administration of several pharmacological medications, for instance, doxorubicin (DOX), an anthracycline that induces either acute or chronic cardiotoxicity [92] (reviewed in [93]). DOX is an effective chemotherapeutic agent widely used for the treatment of different types of cancer, but dose limiting due to its irreversible side effects [94] (reviewed in [93]). It is still unknown whether DOX treatment alters WT1 expression in the heart, however, the effects of its administration in adult mice carrying the cardiomyocyte-*Wt1* deletion have recently been assessed using the transgenic mouse model αMHC^merCremer/+^;Wt1^Flox/Flox^. Picrosirius red staining of tissue sections from control and cardiomyocyte-Wt1 deficient mice showed that both acute and chronic treatment with DOX provoked interstitial fibrosis in the hearts of each group of mice, but the levels of fibrosis were much higher in mice with conditional *Wt1* deletion. This difference was more pronounced in cardiac tissues from animals with chronic DOX administration, suggesting that the lack of WT1 in adult cardiomyocytes worsened the recovery. It has been described that DOX generates mitochondrial damage, and flow cytometry analysis revealed that an acute dose of DOX caused a significant diminution in the mitochondrial load of cardiomyocytes with conditional *Wt1* deletion. Electrocardiographic parameters after chronic DOX treatment demonstrated that mice carrying the *Wt1* deletion exhibited elongated JT, QT, and Tpeak–Tend intervals, and a reduction in the PR interval after 3 months. The proteomic analysis of adult hearts revealed that chronic DOX administration also induced changes in the fatty acid oxidation, in addition to a downregulation in the oxidative phosphorylation and ETC pathways. This result supports the hypothesis that WT1 intervenes in cardiomyocyte metabolism and highlights the need to define its direct role [53].

Mitochondria are the central organelles damaged by both DOX-induced cardiotoxicity and MI (reviewed in [95]). Cardiomyocytes have a high mitochondrial content due to the elevated energy demand of the heart (reviewed in [96]). Consequently, cardiomyocytes result in being the main cell type affected in heart injuries, leading to apoptosis [97]. Fibrosis is another consequence observed in cardiac pathologies [98] (reviewed in [71]), and the additional lack of WT1 in cardiomyocytes from mice treated with acute or chronic DOX administration generates higher levels of interstitial fibrosis, thus the mice take longer to recover [53].

The augmentation of interstitial fibrosis caused by the conditional *Wt1* deletion in adult cardiomyocytes is opposite to the response reported in epicardial cells. Upregulation of WT1 through NF-κβ activation in epicardial cells increases cardiac fibrosis in dystrophic hearts [99]. As WT1 participates in the differentiation of EPDCs into fibroblasts [37], it would be interesting to determine whether this process becomes activated secondary to the loss of WT1 in cardiomyocytes. Additional research is needed to identify the mechanisms that trigger the augmentation of interstitial fibrosis in the absence of the cardiomyocyte-specific WT1 expression, and whether its re-expression in this cell type contributes to a reduction in fibrosis in the ischemic heart.

## 4. The Role of Wt1 in Cardiomyocytes in Heart Regeneration

Progress in cardiovascular medicine has led to the increase in survival rates after MI, however, the subsequent cardiac repair is still insufficient [100]. After MI, the adult mammalian heart activates the scarring process driven by fibroblasts and myofibroblasts in order to replace the damaged myocardium [101] (reviewed in [71]). The fibrotic response is based on the excessive deposition of extracellular matrix (ECM) components. The response consists of two different phases, named replacement and reactive fibrosis. At first, it maintains the integrity of the ventricular myocardium, but then expands from the area of the infarct, therefore affecting the contractility of the heart, and finally, the cardiac output [102] (reviewed in [103]). A pathological remodeling following ischemia can lead to chronic heart disease (CHD), which is associated with hospitalization of the patient and a higher mortality risk [104] (reviewed in [105]).

The fibrotic response triggered in the mammalian heart is due to the reduced capacity of adult cardiomyocytes to proliferate and replace the dead cells [78]. In contrast, the neonatal heart in mammals has a higher regenerative potential, allowing for complete recovery after amputation of the ventricular apex as well as after myocardial ischemia through the proliferation of pre-existing cardiomyocytes [106]. Since this capacity is lost after 7 days of age (P7) in mice, the aim is to understand the mechanisms that orchestrate the correct cardiac repair in neonates, in order to reactivate them and enhance cardiomyocyte renewal in the injured human heart, extending the regenerative window throughout life.

Murine cardiomyocytes lose the ability to regenerate by P7 due to a series of changes that include cell cycle arrest, the shift from hyperplasia to hypertrophy, and the metabolic switch from glycolysis into fatty acid oxidation [106] (reviewed in [107,108]). Transcriptomic analysis of neonatal cardiomyocytes revealed that, between P1 and P14, their maturation involves the hypermethylation of genes associated mainly with DNA replication and cell cycle, explaining the reduction in the proliferation in adult cardiomyocytes [109]. Moreover, it is now known that adult cardiomyocytes from injured hearts lack a transcriptional reversion into a neonatal-like state, while other cardiac cell types, such as CD90+ fibroblasts, exhibit a greater transcriptional plasticity that is associated with an increase in proliferation after MI. In addition, mature cardiomyocytes acquire an epigenetic block that impedes the reentry in the cell cycle needed for cardiac regeneration in adulthood. Leukocytes are another cell type that also undergo a transcriptomic switch, which could be related to the increment of fibrosis in the adult heart following ischemia [110].

Along with cardiomyocyte maturation, many studies have shown that epigenetic modifications are involved in the development of several cardiac diseases in humans including dilated cardiomyopathy, fibrosis, and coronary heart disease [111]. Because of the reversible nature that these transcriptomic modifications exhibit, the preliminary results of studies using animal models for different cardiac pathologies were promising, thanks to the application of epigenetic drugs, also known as “epidrugs” [112] (reviewed in [113]).

A potential application of epidrugs in cardiac repair could be focused on the activation of one particular gene of interest, such as Wt1, although the specificity for the locus activation will be limited, and some off-target effects might be expected. Nevertheless, this approach currently seems to be the most promising for transcription factor targeting in humans.

**Figure 5:**
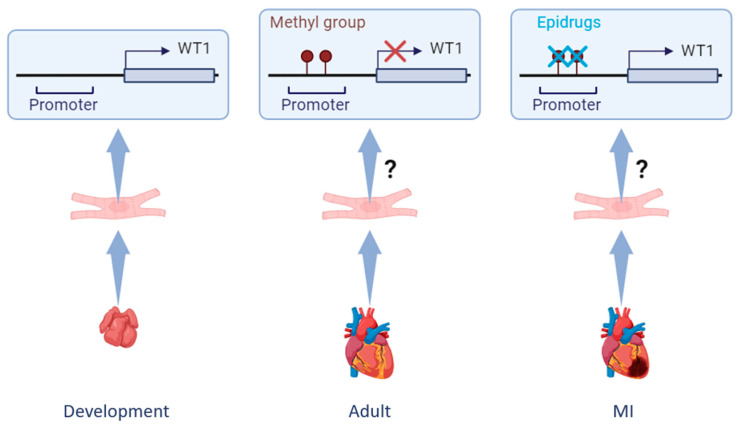
Changes in the methylation levels of WT1 promoter. In the developing heart (**left**), WT1 is highly expressed in embryonic cardiomyocytes, and the WT1 promoter is active. Under homeostatic conditions (**central**), the levels of WT1 are reduced in adult cardiomyocytes, likely due to hypermethylation of its promoter. An interesting approach to enhance the WT1 expression in cardiomyocytes following myocardial infarction (**right**) could be based on the use of “epidrugs”, chemical compounds that can specifically target transcriptional modifications. Therefore, inhibition of DNA methylation in the WT1 promoter would reverse its repression in the adult injured heart. MI: myocardial infarction. Created with BioRender.com.

The methylation of Wt1 promoter and enhancer regions was identified many years ago [114]. While different levels of methylation have been correlated to distinct WT1 expression in several types of cancer, its hypomethylation is associated with more cytoplasmic expression in muscle cells [115]. As previously mentioned, the epicardial reactivation after MI in adult hearts involves the re-expression of embryonic genes, such as WT1, suggesting that this cardiac cell type could exhibit a transcriptional reversion into a less differentiated state. In line with this, chromatin remodeling of Wt1 has been detected in the epicardial activation of the embryonic and adult injured heart, which might be associated with the identification of DNA methylation as one of the main epigenetic modifications that regulates EMT in CHD [116]. For these reasons, an evaluation of the methylation profile of the reactivated epicardial cells would help to confirm whether WT1 re-expression in the injured adult heart is due to a decrease in its methylation level. If confirmed, in addition to the potential identification of an epigenetic biomarker, a promising therapeutic approach could be based on the reduction in the methylation levels of WT1 in adult cardiomyocytes in order to drive its overexpression and shift the cellular profile into a neonatal-like state, which has shown a better response to MI (Figure 5).

In line with this, it has been described that acute exposition to DOX triggers long-term epigenetic modifications in cardiomyocytes. Such modifications are the downregulation of genes that mediate DNA methylation and the upregulation of genes that drive demethylation, overall leading to augmented senescence in cardiomyocytes [117]. The observed increase in genes that participate in active DNA demethylation could be a cardioprotective mechanism, promoting the expression of repressed genes in adult cardiomyocytes. An epigenetic evaluation of cardiomyocytes after chronic DOX administration should be performed in order to confirm whether the same methylation/demethylation profile is maintained or lost through long-term cardiotoxicity.

Li et al. demonstrated that the administration of brain natriuretic peptide (BNP) accelerated the re-expression of WT1 after MI. BNP induced the proliferation of WT1-positive EPDC located in the hypoxic area of the heart, but only in the epicardial and endocardial layers, not in the myocardium [118]. This highlights the proliferative role that WT1 exhibits after infarction, but also the need to identify molecules that induce a more global expression in the heart. Through cardiomyocyte maturation, there is also a variation in the oxygen availability, switching from a hypoxic to an oxygen-rich environment, along with a downregulation of HIF-1α expression in cardiomyocytes from mid-gestation (Figure 4) [119]. However, this physiological regulation seems to worsen the response to myocardial damage in the adult heart, as moderate hypoxia is known to promote cardiac regeneration in zebrafish as well as in adult mice [120]. While an increase in environmental oxygen causes the arrest of the cell cycle in cardiomyocytes, hypoxia can extend their proliferative time window after birth [119]. In humans, severe exposure to low levels of oxygen is related to pathology. However, the controlled exposure to intermittent hypoxic preconditioning (IHP) shows cardioprotective properties and is considered as one of the most promising non-pharmacologic therapies to treat heart failure and coronary heart disease, among other diseases [121] (reviewed in [122]); [123]. Setting a temporally hypoxic environment would avoid the undesired side effects derived from other regenerative approaches that are focused on the long-lasting reversion of cardiomyocytes into a less differentiated state [124]. Some of the negative consequences might be metabolic alterations and tumorigenic levels of cell proliferation.

Therefore, intermittent hypoxic preconditioning seems to be a more appropriate strategy to fight the cardiotoxicity derived from DOX administration. The approach is, however, not suitable for patients that have suffered a myocardial infarction. In Wistar rats, IHP improves cardiac output diminished by DOX administration through the increased expression of SERCA2a and suppressed activation of CaMKII [125]. As hypoxia-induced HIF-1 upregulation activates WT1, this might be one reason for the observed cardioprotective effects of IHP including the ameliorated calcium homeostasis. Further investigation is required to ascertain whether the overexpression of WT1 is a sufficient factor in orchestrating the cardiac regenerative response in the adult heart following ischemic injury or cardiotoxic events. Although this approach appears feasible in transgenic animal models, the implementation of gene therapy in humans is constrained by significant ethical considerations. Furthermore, the delivery strategy specifically to the heart after myocardial infarction and the potential for off-target effects of the constructs present significant challenges for potential clinical applications. Additionally, as WT1 is an important regulator of tumor growth [15], safety concerns associated with a generalized overexpression must be considered. Consequently, the identification of short-lived epigenetic drugs modulating WT1 expression may offer a more promising avenue, although limited specificity could be anticipated, as previously discussed.

## 5. Conclusions

WT1 is commonly known as an epicardial marker based on its high levels of expression during heart development, where it drives epithelial to mesenchymal transition, but as described here, it is also expressed in other cardiac cell types such as cardiomyocytes. The fact that it is not only found in the embryonic heart, but continues to be expressed, although to a lesser extent, in the adult heart, and that its expression strongly increases after ischemic damage suggests that WT1 is essential for cardiac development and might favor adult cardiac repair. The strong cardiomyocytic WT1 re-expression after myocardial infarction supports the idea that it could have a cardioprotective role. This is further supported by the finding that hearts from adult mice with conditional *Wt1* deletion suffered metabolic alterations, along with interstitial fibrosis and hypertrophy, and also displayed a worse outcome in the case of cardiotoxic doxorubicin treatment. Further research is needed to clarify the mechanisms leading to Wt1 re-expression in adult cardiomyocytes such as hypoxia and possibly changes in its methylation profile. In addition, it needs to be clarified whether WT1 re-expression is related to an induction of proliferation in order to compensate for the cardiomyocyte loss after myocardial infarction. As WT1 seems to be implicated in cardiac calcium regulation, it might prevent cardiomyocytic Ca^2+^ intracellular accumulation, suggesting that WT1 might be involved in the reduction in the elevated levels of calcium after myocardial infarction. In summary, based on the essential expression of WT1 in the embryonic heart and its reactivation after damage, it should be considered as one of the genetic targets that could induce regenerative responses in cardiomyocytes after ischemia. However, further clarification of the processes that regulate WT1 in cardiomyocytes and the consequences of its activation for cardiac repair are required before a potential therapeutic approach can be considered.

## Data Availability

Not applicable.

## Abbreviations

αMHC	α-Myosin heavy chain
BNP	Brain natriuretic peptide
Ca^2+^	Calcium
CaMKIIẟ	Calcium/calmodulin-dependent protein kinase II ẟ
CHD	Chronic heart disease
DOX	Doxorubicin
E	Embryonic day
ECC	Excitation-contraction coupling
ECM	Extracellular matrix
EMT	Epithelial-to-mesenchymal transition
EPDCs	Epicardial-derived cells
ETC	Electron transport chain
EYFP	Enhanced yellow fluorescent protein
GFP	Green fluorescent protein
HF	Heart failure
HIF1	Hypoxia inducible factor-1
IHP	Intermittent hypoxic preconditioning
Kcnk2	Trek-1
KTS	Lysine, threonine, serine
LAD	Ligation of the left anterior descending coronary artery
Mer	Mutant estrogen receptor
MI	Myocardial infarction
NC-RNA	Non-coding RNA
P	Postnatal day
PTMs	Post-translational modifications
Raldh	Retinaldehyde-dehydrogenase
SERCA	Sarcoplasmic/endoplasmic reticulum Ca^2+^ ATPase
ShRNA	Short hairpin RNA
SHRs	Spontaneously hypertensive rats
Snai1	Snail
SOCE	Store-operated Ca^2+^ entry
STIM1	Stromal interaction molecule 1
Tbx18	T-box transcription factor 18
VDR	Vitamin D receptors
WT	Wilms’ tumor
WT1	Wilms’ tumor suppressor 1

## References

[1] Haber D.A., Buckler A.J., Glaser T., Call K.M., Pelletier J., Sohn R.L., Douglass E.C., Housman D.E. (1990). An internal deletion within an 11p13 zinc finger gene contributes to the development of Wilms’ tumor. Cell.

[2] Gessler M., König A., Bruns G.A. (1992). The genomic organization and expression of the WT1 gene. Genomics.

[3] Little M.H., Prosser J., Condie A., Smith P.J., Van Heyningen V., Hastie N.D. (1992). Zinc finger point mutations within the WT1 gene in Wilms tumor patients. Proc. Natl. Acad. Sci. USA.

[4] Buckler A.J., Pelletier J., Haber D.A., Glaser T., Housman D.E. (1991). Isolation, characterization, and expression of the murine Wilms’ tumor gene (WT1) during kidney development. Mol. Cell. Biol..

[5] Huff V., Miwa H., Haber D.A., Call K.M., Housman D., Strong L.C., Saunders G.F. (1991). Evidence for WT1 as a Wilms tumor (WT) gene: Intragenic germinal deletion in bilateral WT. Am. J. Hum. Genet..

[6] Little M., Wells C. (1997). A clinical overview of WT1 gene mutations. Hum. Mutat..

[7] Rivera M.N., Haber D.A. (2005). Wilms’ tumour: Connecting tumorigenesis and organ development in the kidney. Nat. Rev. Cancer.

[8] Miwa H., Tomlinson G.E., Timmons C.F., Huff V., Cohn S.L., Strong L.C., Saunders G.F. (1992). RNA expression of the WT1 gene in Wilms’ tumors in relation to histology. J. Natl. Cancer Inst..

[9] Oji Y., Miyoshi S., Maeda H., Hayashi S., Tamaki H., Nakatsuka S., Yao M., Takahashi E., Nakano Y., Hirabayashi H. (2002). Overexpression of the Wilms’ tumor gene WT1 in de novo lung cancers. Int. J. Cancer.

[10] Oji Y., Ogawa H., Tamaki H., Oka Y., Tsuboi A., Kim E.H., Soma T., Tatekawa T., Kawakami M., Asada M. (1999). Expression of the Wilms’ tumor gene WT1 in solid tumors and its involvement in tumor cell growth. Jpn. J. Cancer Res..

[11] Wagner N., Panelos J., Massi D., Wagner K.D. (2008). The Wilms’ tumor suppressor WT1 is associated with melanoma proliferation. Pflug. Arch..

[12] Sugiyama H. (2010). WT1 (Wilms’ tumor gene 1): Biology and cancer immunotherapy. Jpn. J. Clin. Oncol..

[13] Yoon J.H., Kim H.J., Kwak D.H., Park S.S., Jeon Y.W., Lee S.E., Cho B.S., Eom K.S., Kim Y.J., Lee S. (2017). High WT1 expression is an early predictor for relapse in patients with acute promyelocytic leukemia in first remission with negative PML-RARa after anthracycline-based chemotherapy: A single-center cohort study. J. Hematol. Oncol..

[14] Maslak P.G., Dao T., Bernal Y., Chanel S.M., Zhang R., Frattini M., Rosenblat T., Jurcic J.G., Brentjens R.J., Arcila M.E. (2018). Phase 2 trial of a multivalent WT1 peptide vaccine (galinpepimut-S) in acute myeloid leukemia. Blood Adv..

[15] Wagner K.D., Cherfils-Vicini J., Hosen N., Hohenstein P., Gilson E., Hastie N.D., Michiels J.F., Wagner N. (2014). The Wilms’ tumour suppressor Wt1 is a major regulator of tumour angiogenesis and progression. Nat. Commun..

[16] Cebinelli G.C., DE Sousa Pereira N., Sena M.M., DE Oliveira C.E., Fujita T.C., DA Rocha S.P., DE Abreu Oliveira F.J., Marinello P.C., Watanabe M.A. (2016). Immunotherapy in Acute Leukemias: Implications and Perspectives Using Wt1 Antigen. Anticancer. Res..

[17] Sugiyama H. (2002). Cancer immunotherapy targeting WT1 protein. Int. J. Hematol..

[18] Van Driessche A., Berneman Z.N., Van Tendeloo V.F. (2012). Active specific immunotherapy targeting the Wilms’ tumor protein 1 (WT1) for patients with hematological malignancies and solid tumors: Lessons from early clinical trials. Oncologist.

[19] Dao T., Xiong G., Mun S.S., Meyerberg J., Korontsvit T., Xiang J., Cui Z., Chang A.Y., Jarvis C., Cai W. (2024). A dual-receptor T-cell platform with Ab-TCR and costimulatory receptor achieves specificity and potency against AML. Blood.

[20] Wagner K.D., Wagner N., Schedl A. (2003). The complex life of WT1. J. Cell Sci..

[21] Morrison A.A., Viney R.L., Ladomery M.R. (2008). The post-transcriptional roles of WT1, a multifunctional zinc-finger protein. Biochim. Biophys. Acta.

[22] Toska E., Roberts S.G. (2014). Mechanisms of transcriptional regulation by WT1 (Wilms’ tumour 1). Biochem. J..

[23] Hastie N.D. (2017). Wilms’ tumour 1 (WT1) in development, homeostasis and disease. Development.

[24] Larsson S.H., Charlieu J.P., Miyagawa K., Engelkamp D., Rassoulzadegan M., Ross A., Cuzin F., van Heyningen V., Hastie N.D. (1995). Subnuclear localization of WT1 in splicing or transcription factor domains is regulated by alternative splicing. Cell.

[25] Haber D.A., Sohn R.L., Buckler A.J., Pelletier J., Call K.M., Housman D.E. (1991). Alternative splicing and genomic structure of the Wilms tumor gene WT1. Proc. Natl. Acad. Sci. USA.

[26] Niksic M., Slight J., Sanford J.R., Caceres J.F., Hastie N.D. (2004). The Wilms’ tumour protein (WT1) shuttles between nucleus and cytoplasm and is present in functional polysomes. Hum. Mol. Genet..

[27] Kreidberg J.A., Sariola H., Loring J.M., Maeda M., Pelletier J., Housman D., Jaenisch R. (1993). WT-1 is required for early kidney development. Cell.

[28] Martínez-Estrada O.M., Lettice L.A., Essafi A., Guadix J.A., Slight J., Velecela V., Hall E., Reichmann J., Devenney P.S., Hohenstein P. (2010). Wt1 is required for cardiovascular progenitor cell formation through transcriptional control of Snail and E-cadherin. Nat. Genet..

[29] Díaz Del Moral S., Barrena S., Hernández-Torres F., Aránega A., Villaescusa J.M., Gómez Doblas J.J., Franco D., Jiménez-Navarro M., Muñoz-Chápuli R., Carmona R. (2021). Deletion of the Wilms’ Tumor Suppressor Gene in the Cardiac Troponin-T Lineage Reveals Novel Functions of WT1 in Heart Development. Front. Cell Dev. Biol..

[30] Wagner N., Wagner K.D. (2021). Every Beat You Take-The Wilms’ Tumor Suppressor WT1 and the Heart. Int. J. Mol. Sci..

[31] Wagner N., Ninkov M., Vukolic A., Cubukcuoglu Deniz G., Rassoulzadegan M., Michiels J.F., Wagner K.D. (2021). Implications of the Wilms’ Tumor Suppressor Wt1 in Cardiomyocyte Differentiation. Int. J. Mol. Sci..

[32] Armstrong J.F., Pritchard-Jones K., Bickmore W.A., Hastie N.D., Bard J.B. (1993). The expression of the Wilms’ tumour gene, WT1, in the developing mammalian embryo. Mech. Dev..

[33] Moore A.W., McInnes L., Kreidberg J., Hastie N.D., Schedl A. (1999). YAC complementation shows a requirement for Wt1 in the development of epicardium, adrenal gland and throughout nephrogenesis. Development.

[34] Zhou B., Ma Q., Rajagopal S., Wu S.M., Domian I., Rivera-Feliciano J., Jiang D., von Gise A., Ikeda S., Chien K.R. (2008). Epicardial progenitors contribute to the cardiomyocyte lineage in the developing heart. Nature.

[35] Vicente-Steijn R., Scherptong R.W., Kruithof B.P., Duim S.N., Goumans M.J., Wisse L.J., Zhou B., Pu W.T., Poelmann R.E., Schalij M.J. (2015). Regional differences in WT-1 and Tcf21 expression during ventricular development: Implications for myocardial compaction. PLoS ONE.

[36] von Gise A., Zhou B., Honor L.B., Ma Q., Petryk A., Pu W.T. (2011). WT1 regulates epicardial epithelial to mesenchymal transition through β-catenin and retinoic acid signaling pathways. Dev. Biol..

[37] Zhou B., Honor L.B., He H., Ma Q., Oh J.H., Butterfield C., Lin R.Z., Melero-Martin J.M., Dolmatova E., Duffy H.S. (2011). Adult mouse epicardium modulates myocardial injury by secreting paracrine factors. J. Clin. Investig..

[38] Pérez-Pomares J.M., Phelps A., Sedmerova M., Carmona R., González-Iriarte M., Muñoz-Chápuli R., Wessels A. (2002). Experimental studies on the spatiotemporal expression of WT1 and RALDH2 in the embryonic avian heart: A model for the regulation of myocardial and valvuloseptal development by epicardially derived cells (EPDCs). Dev. Biol..

[39] Wessels A., van den Hoff M.J., Adamo R.F., Phelps A.L., Lockhart M.M., Sauls K., Briggs L.E., Norris R.A., van Wijk B., Perez-Pomares J.M. (2012). Epicardially derived fibroblasts preferentially contribute to the parietal leaflets of the atrioventricular valves in the murine heart. Dev. Biol..

[40] Cai C.L., Martin J.C., Sun Y., Cui L., Wang L., Ouyang K., Yang L., Bu L., Liang X., Zhang X. (2008). A myocardial lineage derives from Tbx18 epicardial cells. Nature.

[41] Christoffels V.M., Grieskamp T., Norden J., Mommersteeg M.T., Rudat C., Kispert A. (2009). Tbx18 and the fate of epicardial progenitors. Nature.

[42] Tyser R.C.V., Ibarra-Soria X., McDole K., Arcot Jayaram S., Godwin J., van den Brand T.A.H., Miranda A.M.A., Scialdone A., Keller P.J., Marioni J.C. (2021). Characterization of a common progenitor pool of the epicardium and myocardium. Science.

[43] Guadix J.A., Ruiz-Villalba A., Lettice L., Velecela V., Muñoz-Chápuli R., Hastie N.D., Pérez-Pomares J.M., Martínez-Estrada O.M. (2011). Wt1 controls retinoic acid signalling in embryonic epicardium through transcriptional activation of Raldh2. Development.

[44] Da Silva F., Jian Motamedi F., Weerasinghe Arachchige L.C., Tison A., Bradford S.T., Lefebvre J., Dolle P., Ghyselinck N.B., Wagner K.D., Schedl A. (2021). Retinoic acid signaling is directly activated in cardiomyocytes and protects mouse hearts from apoptosis after myocardial infarction. eLife.

[45] Wagner N., Wagner K.D., Theres H., Englert C., Schedl A., Scholz H. (2005). Coronary vessel development requires activation of the TrkB neurotrophin receptor by the Wilms’ tumor transcription factor Wt1. Genes. Dev..

[46] Rudat C., Kispert A. (2012). Wt1 and epicardial fate mapping. Circ. Res..

[47] Duim S.N., Kurakula K., Goumans M.J., Kruithof B.P. (2015). Cardiac endothelial cells express Wilms’ tumor-1: Wt1 expression in the developing, adult and infarcted heart. J. Mol. Cell Cardiol..

[48] Wagner N., Morrison H., Pagnotta S., Michiels J.F., Schwab Y., Tryggvason K., Schedl A., Wagner K.D. (2011). The podocyte protein nephrin is required for cardiac vessel formation. Hum. Mol. Genet..

[49] Scholz H., Wagner K.D., Wagner N. (2009). Role of the Wilms’ tumour transcription factor, Wt1, in blood vessel formation. Pflug. Arch..

[50] Cano E., Carmona R., Ruiz-Villalba A., Rojas A., Chau Y.Y., Wagner K.D., Wagner N., Hastie N.D., Muñoz-Chápuli R., Pérez-Pomares J.M. (2016). Extracardiac septum transversum/proepicardial endothelial cells pattern embryonic coronary arterio-venous connections. Proc. Natl. Acad. Sci. USA.

[51] Ramiro-Pareta M., Müller-Sánchez C., Portella-Fortuny R., Soler-Botija C., Torres-Cano A., Esteve-Codina A., Bayés-Genís A., Reina M., Soriano F.X., Montanez E. (2023). Endothelial deletion of Wt1 disrupts coronary angiogenesis and myocardium development. Development.

[52] Smart N., Bollini S., Dubé K.N., Vieira J.M., Zhou B., Davidson S., Yellon D., Riegler J., Price A.N., Lythgoe M.F. (2011). De novo cardiomyocytes from within the activated adult heart after injury. Nature.

[53] Díaz Del Moral S., Benaouicha M., Villa Del Campo C., Torres M., Wagner N., Wagner K.D., Muñoz-Chápuli R., Carmona R. (2023). Cardiomyocyte-Specific Wt1 Is Involved in Cardiac Metabolism and Response to Damage. J. Cardiovasc. Dev. Dis..

[54] Marques I.J., Ernst A., Arora P., Vianin A., Hetke T., Sanz-Morejón A., Naumann U., Odriozola A., Langa X., Andrés-Delgado L. (2022). Wt1 transcription factor impairs cardiomyocyte specification and drives a phenotypic switch from myocardium to epicardium. Development.

[55] Hosen N., Shirakata T., Nishida S., Yanagihara M., Tsuboi A., Kawakami M., Oji Y., Oka Y., Okabe M., Tan B. (2007). The Wilms’ tumor gene WT1-GFP knock-in mouse reveals the dynamic regulation of WT1 expression in normal and leukemic hematopoiesis. Leukemia.

[56] Srinivas S., Watanabe T., Lin C.S., William C.M., Tanabe Y., Jessell T.M., Costantini F. (2001). Cre reporter strains produced by targeted insertion of EYFP and ECFP into the ROSA26 locus. BMC Dev. Biol..

[57] Rawat H., Kornherr J., Zawada D., Bakhshiyeva S., Kupatt C., Laugwitz K.L., Bähr A., Dorn T., Moretti A., Nowak-Imialek M. (2023). Recapitulating porcine cardiac development. Front. Cell Dev. Biol..

[58] Jinton H., Sopasakis V.R., Sjölin L., Oldfors A., Jeppsson A., Oras J., Wernbom M., Vukusic K. (2024). Global ischemia induces stemness and dedifferentiation in human adult cardiomyocytes after cardiac arrest. Sci. Rep..

[59] Jiao K., Kulessa H., Tompkins K., Zhou Y., Batts L., Baldwin H.S., Hogan B.L. (2003). An essential role of Bmp4 in the atrioventricular septation of the mouse heart. Genes. Dev..

[60] Ritchie M.F., Yue C., Zhou Y., Houghton P.J., Soboloff J. (2010). Wilms tumor suppressor 1 (WT1) and early growth response 1 (EGR1) are regulators of STIM1 expression. J. Biol. Chem..

[61] Fozzard H.A. (1977). Heart: Excitation-contraction coupling. Annu. Rev. Physiol..

[62] Liou J., Kim M.L., Heo W.D., Jones J.T., Myers J.W., Ferrell J.E., Meyer T. (2005). STIM is a Ca^2+^ sensor essential for Ca^2+^-store-depletion-triggered Ca^2+^ influx. Curr. Biol..

[63] Rosenberg P., Zhang H., Bryson V.G., Wang C. (2021). SOCE in the cardiomyocyte: The secret is in the chambers. Pflug. Arch..

[64] Edman C.F., Schulman H. (1994). Identification and characterization of delta B-CaM kinase and delta C-CaM kinase from rat heart, two new multifunctional Ca^2+^/calmodulin-dependent protein kinase isoforms. Biochim. Biophys. Acta.

[65] Walker C., Rutten F., Yuan X., Pass H., Mew D.M., Everitt J. (1994). Wilms’ tumor suppressor gene expression in rat and human mesothelioma. Cancer Res..

[66] Sohal D.S., Nghiem M., Crackower M.A., Witt S.A., Kimball T.R., Tymitz K.M., Penninger J.M., Molkentin J.D. (2001). Temporally regulated and tissue-specific gene manipulations in the adult and embryonic heart using a tamoxifen-inducible Cre protein. Circ. Res..

[67] Chau Y.Y., Brownstein D., Mjoseng H., Lee W.C., Buza-Vidas N., Nerlov C., Jacobsen S.E., Perry P., Berry R., Thornburn A. (2011). Acute multiple organ failure in adult mice deleted for the developmental regulator Wt1. PLoS Genet..

[68] Roger V.L. (2013). Epidemiology of heart failure. Circ. Res..

[69] Laflamme M.A., Murry C.E. (2005). Regenerating the heart. Nat. Biotechnol..

[70] Fishbein M.C., Maclean D., Maroko P.R. (1978). Experimental myocardial infarction in the rat: Qualitative and quantitative changes during pathologic evolution. Am. J. Pathol..

[71] Talman V., Ruskoaho H. (2016). Cardiac fibrosis in myocardial infarction-from repair and remodeling to regeneration. Cell Tissue Res..

[72] Bugger H., Pfeil K. (2020). Mitochondrial ROS in myocardial ischemia reperfusion and remodeling. Biochim. Biophys. Acta Mol. Basis Dis..

[73] Wagner K.D., Wagner N., Bondke A., Nafz B., Flemming B., Theres H., Scholz H. (2002). The Wilms’ tumor suppressor Wt1 is expressed in the coronary vasculature after myocardial infarction. FASEB J..

[74] van Wijk B., Gunst Q.D., Moorman A.F., van den Hoff M.J. (2012). Cardiac regeneration from activated epicardium. PLoS ONE.

[75] Bollini S., Vieira J.M., Howard S., Dubè K.N., Balmer G.M., Smart N., Riley P.R. (2014). Re-activated adult epicardial progenitor cells are a heterogeneous population molecularly distinct from their embryonic counterparts. Stem Cells Dev..

[76] Smart N., Risebro C.A., Melville A.A., Moses K., Schwartz R.J., Chien K.R., Riley P.R. (2007). Thymosin beta4 induces adult epicardial progenitor mobilization and neovascularization. Nature.

[77] Zhou B., Honor L.B., Ma Q., Oh J.H., Lin R.Z., Melero-Martin J.M., von Gise A., Zhou P., Hu T., He L. (2012). Thymosin beta 4 treatment after myocardial infarction does not reprogram epicardial cells into cardiomyocytes. J. Mol. Cell. Cardiol..

[78] Hsieh P.C., Segers V.F., Davis M.E., MacGillivray C., Gannon J., Molkentin J.D., Robbins J., Lee R.T. (2007). Evidence from a genetic fate-mapping study that stem cells refresh adult mammalian cardiomyocytes after injury. Nat. Med..

[79] Hulot J.S., Fauconnier J., Ramanujam D., Chaanine A., Aubart F., Sassi Y., Merkle S., Cazorla O., Ouillé A., Dupuis M. (2011). Critical role for stromal interaction molecule 1 in cardiac hypertrophy. Circulation.

[80] Kirchhefer U., Schmitz W., Scholz H., Neumann J. (1999). Activity of cAMP-dependent protein kinase and Ca^2+^/calmodulin-dependent protein kinase in failing and nonfailing human hearts. Cardiovasc. Res..

[81] Giorgi C., Romagnoli A., Pinton P., Rizzuto R. (2008). Ca^2+^ signaling, mitochondria and cell death. Curr. Mol. Med..

[82] Agyapong E.D., Pedriali G., Ramaccini D., Bouhamida E., Tremoli E., Giorgi C., Pinton P., Morciano G. (2024). Calcium signaling from sarcoplasmic reticulum and mitochondria contact sites in acute myocardial infarction. J. Transl. Med..

[83] Wagner K.D., Wagner N., Wellmann S., Schley G., Bondke A., Theres H., Scholz H. (2003). Oxygen-regulated expression of the Wilms’ tumor suppressor Wt1 involves hypoxia-inducible factor-1 (HIF-1). FASEB J..

[84] Sato T., Takeda N. (2023). The roles of HIF-1α signaling in cardiovascular diseases. J. Cardiol..

[85] Tao J., Doughman Y., Yang K., Ramirez-Bergeron D., Watanabe M. (2013). Epicardial HIF signaling regulates vascular precursor cell invasion into the myocardium. Dev. Biol..

[86] Krueger K., Catanese L., Sciesielski L.K., Kirschner K.M., Scholz H. (2019). Deletion of an intronic HIF-2α binding site suppresses hypoxia-induced WT1 expression. Biochim. Biophys. Acta Gene Regul. Mech..

[87] Scragg R., Jackson R., Holdaway I.M., Lim T., Beaglehole R. (1990). Myocardial infarction is inversely associated with plasma 25-hydroxyvitamin D3 levels: A community-based study. Int. J. Epidemiol..

[88] Beveridge L.A., Witham M.D. (2013). Vitamin D and the cardiovascular system. Osteoporos. Int..

[89] Wong M.S., Leisegang M.S., Kruse C., Vogel J., Schürmann C., Dehne N., Weigert A., Herrmann E., Brüne B., Shah A.M. (2014). Vitamin D promotes vascular regeneration. Circulation.

[90] Barbarawi M., Kheiri B., Zayed Y., Barbarawi O., Dhillon H., Swaid B., Yelangi A., Sundus S., Bachuwa G., Alkotob M.L. (2019). Vitamin D Supplementation and Cardiovascular Disease Risks in More Than 83 000 Individuals in 21 Randomized Clinical Trials: A Meta-analysis. JAMA Cardiol..

[91] Wagner K.D., Wagner N., Sukhatme V.P., Scholz H. (2001). Activation of vitamin D receptor by the Wilms’ tumor gene product mediates apoptosis of renal cells. J. Am. Soc. Nephrol..

[92] Steinherz L., Steinherz P. (1991). Delayed cardiac toxicity from anthracycline therapy. Pediatrician.

[93] Minotti G., Menna P., Salvatorelli E., Cairo G., Gianni L. (2004). Anthracyclines: Molecular advances and pharmacologic developments in antitumor activity and cardiotoxicity. Pharmacol. Rev..

[94] Weiss R.B. (1992). The anthracyclines: Will we ever find a better doxorubicin?. Semin. Oncol..

[95] Wallace K.B. (2003). Doxorubicin-induced cardiac mitochondrionopathy. Pharmacol. Toxicol..

[96] Ventura-Clapier R., Garnier A., Veksler V. (2004). Energy metabolism in heart failure. J. Physiol..

[97] Solem L.E., Heller L.J., Wallace K.B. (1996). Dose-dependent increase in sensitivity to calcium-induced mitochondrial dysfunction and cardiomyocyte cell injury by doxorubicin. J. Mol. Cell. Cardiol..

[98] Caulfield J.B., Bittner V. (1988). Cardiac matrix alterations induced by adriamycin. Am. J. Pathol..

[99] Guo Z., Geng M., Huang Y., Han G., Jing R., Lin C., Zhang X., Zhang M., Fan G., Wang F. (2022). Upregulation of Wilms’ Tumor 1 in epicardial cells increases cardiac fibrosis in dystrophic mice. Cell Death Differ..

[100] Quijada P., Trembley M.A., Small E.M. (2020). The Role of the Epicardium During Heart Development and Repair. Circ. Res..

[101] Sutton M.G., Sharpe N. (2000). Left ventricular remodeling after myocardial infarction: Pathophysiology and therapy. Circulation.

[102] Thiedemann K.U., Holubarsch C., Medugorac I., Jacob R. (1983). Connective tissue content and myocardial stiffness in pressure overload hypertrophy. A combined study of morphologic, morphometric, biochemical, and mechanical parameters. Basic Res. Cardiol..

[103] Rockey D.C., Bell P.D., Hill J.A. (2015). Fibrosis--a common pathway to organ injury and failure. N. Engl. J. Med..

[104] Bolognese L., Neskovic A.N., Parodi G., Cerisano G., Buonamici P., Santoro G.M., Antoniucci D. (2002). Left ventricular remodeling after primary coronary angioplasty: Patterns of left ventricular dilation and long-term prognostic implications. Circulation.

[105] Snipelisky D., Chaudhry S.P., Stewart G.C. (2019). The Many Faces of Heart Failure. Card. Electrophysiol. Clin..

[106] Porrello E.R., Mahmoud A.I., Simpson E., Hill J.A., Richardson J.A., Olson E.N., Sadek H.A. (2011). Transient regenerative potential of the neonatal mouse heart. Science.

[107] Lopaschuk G.D., Jaswal J.S. (2010). Energy metabolic phenotype of the cardiomyocyte during development, differentiation, and postnatal maturation. J. Cardiovasc. Pharmacol..

[108] Karbassi E., Fenix A., Marchiano S., Muraoka N., Nakamura K., Yang X., Murry C.E. (2020). Cardiomyocyte maturation: Advances in knowledge and implications for regenerative medicine. Nat. Rev. Cardiol..

[109] Sim C.B., Ziemann M., Kaspi A., Harikrishnan K.N., Ooi J., Khurana I., Chang L., Hudson J.E., El-Osta A., Porrello E.R. (2015). Dynamic changes in the cardiac methylome during postnatal development. FASEB J..

[110] Quaife-Ryan G.A., Sim C.B., Ziemann M., Kaspi A., Rafehi H., Ramialison M., El-Osta A., Hudson J.E., Porrello E.R. (2017). Multicellular Transcriptional Analysis of Mammalian Heart Regeneration. Circulation.

[111] Movassagh M., Choy M.K., Knowles D.A., Cordeddu L., Haider S., Down T., Siggens L., Vujic A., Simeoni I., Penkett C. (2011). Distinct epigenomic features in end-stage failing human hearts. Circulation.

[112] Xiao D., Dasgupta C., Chen M., Zhang K., Buchholz J., Xu Z., Zhang L. (2014). Inhibition of DNA methylation reverses norepinephrine-induced cardiac hypertrophy in rats. Cardiovasc. Res..

[113] Gorica E., Mohammed S.A., Ambrosini S., Calderone V., Costantino S., Paneni F. (2022). Epi-Drugs in Heart Failure. Front. Cardiovasc. Med..

[114] Laux D.E., Curran E.M., Welshons W.V., Lubahn D.B., Huang T.H. (1999). Hypermethylation of the Wilms’ tumor suppressor gene CpG island in human breast carcinomas. Breast Cancer Res. Treat..

[115] Kaneuchi M., Sasaki M., Tanaka Y., Shiina H., Yamada H., Yamamoto R., Sakuragi N., Enokida H., Verma M., Dahiya R. (2005). WT1 and WT1-AS genes are inactivated by promoter methylation in ovarian clear cell adenocarcinoma. Cancer.

[116] Vieira J.M., Howard S., Villa Del Campo C., Bollini S., Dubé K.N., Masters M., Barnette D.N., Rohling M., Sun X., Hankins L.E. (2017). BRG1-SWI/SNF-dependent regulation of the Wt1 transcriptional landscape mediates epicardial activity during heart development and disease. Nat. Commun..

[117] Robinson E.L., Ameri P., Delrue L., Vanderheyden M., Bartunek J., Altieri P., Heymans S., Heggermont W.A. (2023). Differential expression of epigenetic modifiers in early and late cardiotoxic heart failure reveals DNA methylation as a key regulator of cardiotoxicity. Front. Cardiovasc. Med..

[118] Li N., Rignault-Clerc S., Bielmann C., Bon-Mathier A.C., Déglise T., Carboni A., Ducrest M., Rosenblatt-Velin N. (2020). Increasing heart vascularisation after myocardial infarction using brain natriuretic peptide stimulation of endothelial and WT1. eLife.

[119] Puente B.N., Kimura W., Muralidhar S.A., Moon J., Amatruda J.F., Phelps K.L., Grinsfelder D., Rothermel B.A., Chen R., Garcia J.A. (2014). The oxygen-rich postnatal environment induces cardiomyocyte cell-cycle arrest through DNA damage response. Cell.

[120] Jopling C., Suñé G., Faucherre A., Fabregat C., Izpisua Belmonte J.C. (2012). Hypoxia induces myocardial regeneration in zebrafish. Circulation.

[121] Sanchis-Gomar F., Viña J., Lippi G. (2012). Intermittent hypobaric hypoxia applicability in myocardial infarction prevention and recovery. J. Cell. Mol. Med..

[122] Navarrete-Opazo A., Mitchell G.S. (2014). Therapeutic potential of intermittent hypoxia: A matter of dose. Am. J. Physiol. Regul. Integr. Comp. Physiol..

[123] Cai M., Chen X., Shan J., Yang R., Guo Q., Bi X., Xu P., Shi X., Chu L., Wang L. (2021). Intermittent Hypoxic Preconditioning: A Potential New Powerful Strategy for COVID-19 Rehabilitation. Front. Pharmacol..

[124] Chen Y., Lüttmann F.F., Schoger E., Schöler H.R., Zelarayán L.C., Kim K.P., Haigh J.J., Kim J., Braun T. (2021). Reversible reprogramming of cardiomyocytes to a fetal state drives heart regeneration in mice. Science.

[125] Galis P., Bartosova L., Farkasova V., Szobi A., Horvath C., Kovacova D., Adameova A., Rajtik T. (2023). Intermittent Hypoxic Preconditioning Plays a Cardioprotective Role in Doxorubicin-Induced Cardiomyopathy. Cardiovasc. Toxicol..

